# Flavonoids target different molecules of autophagic and metastatic pathways in cancer cells

**DOI:** 10.1186/s12935-023-02960-4

**Published:** 2023-06-12

**Authors:** Aysooda Hosseinzadeh, Faezeh Poursoleiman, Akram Naghdipour Biregani, Ahmad Esmailzadeh

**Affiliations:** 1 Department of Immunology, Faculty of Medicine, Shahid Sadoughi University of Medical Sciences, Yazd, Iran; 2 Department of Cellular and Molecular Nutrition, Shahid Beheshti University of Medical Sciences, Tehran, Iran; 3 Department of Nutrition, School of Health, Shahid Sadoughi University of Medical Scinences, Yazd, Iran; 4 Students’ Scientific Center, Tehran University of Medical Sciences, Tehran, Iran; 5 Endocrinology and Metabolism Research Center, Endocrinology and Metabolism Clinical Sciences Institute, Tehran University of Medical Sciences, Tehran, Islamic Republic of Iran; 6 Food Security Research Center, Department of Community Nutrition, School of Nutrition and Food Science, Isfahan University of Medical Sciences, Isfahan, Iran

**Keywords:** Autophagy, Metastasis, Anoikis, Cancer, Drug resistance

## Abstract

Despite the success of cancer therapy, it has encountered a major obstacle due to the complicated nature of cancer, namely resistance. The recurrence and metastasis of cancer occur when anti-cancer therapeutic agents fail to eradicate all cancer cells. Cancer therapy aims to find the best agent that targets all cancer cells, including those sensitive or resistant to treatment. Flavonoids, natural products from our diet, show anti-cancer effects in different studies. They can inhibit metastasis and the recurrence of cancers. This review discusses metastasis, autophagy, anoikis in cancer cells, and their dynamic relationship. We present evidence that flavonoids can block metastasis and induce cell death in cancer cells. Our research suggests that flavonoids can serve as potential therapeutic agents in cancer therapy.

**Supplementary Information:**

The online version contains supplementary material available at 10.1186/s12935-023-02960-4.

## Background

Cancer is a leading cause of mortality worldwide. It is a complex disease that requires an in-depth understanding of its mechanisms to treat it successfully. Our article discusses mechanisms involved in cancer cells, such as metastasis, autophagy, and anoikis. Following this, we examine how flavonoids may influence these mechanisms. Then, to support our hypothesis that flavonoids serve as potential anti-cancer therapeutic agents, we examine flavonoid results in human studies.

To begin with, we should provide a brief overview of flavonoids. What are these natural products? Flavonoids are a group of secondary metabolites in plants that have recently captured the interest of researchers. They are responsible for color, fragrance, and flavor characteristics and protect plants from stressful situations. They are hydroxylated phenolic substances with two benzene rings (A and B) and a heterocyclic pyran ring (C) in a 15-carbon (C6–C3–C6) skeleton. Considering their chemical structure, there are 6 major groups of flavonoids: flavones (e.g., apigenin and luteolin), flavonols (e.g., quercetin and kaempferol), flavanols (e.g., catechin and epicatechin), flavanones (e.g., hesperetin and naringenin), anthocyanidins (e.g., Cyanidin and Delphinidin) and isoflavonoids (e.g., daidzein and genistein) [1–4].

The following sections examine the molecular mechanisms underlying metastasis and autophagy and how flavonoids affect them.

## Metastasis

**Fig. 1:**
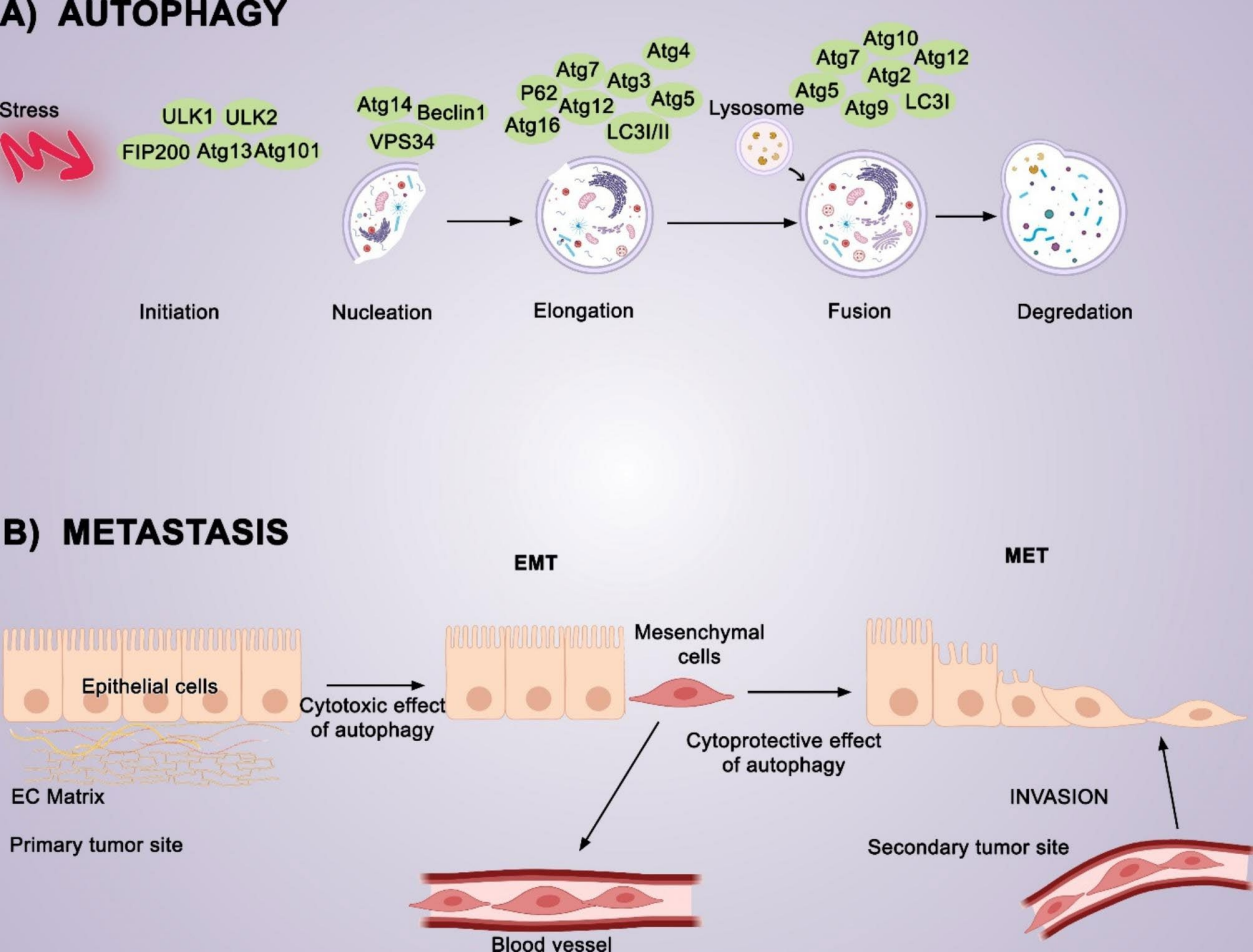
Autophagy and metastasis processes in details **(A) Autophagy**: In a molecular context, autophagy begins with the formation of the ULK1/2 and Atg13/101 complex, followed by the engagement of Beclin1 and Atg14. The elongation step is completed with the recruitment of other Atgs and LC1/II. In the fusion step, the lysosome is added to the preexisting autophagosome, which results in the degradation of autophagosome content. **(B) Metastasis**: Epithelial tissue contains individual epithelial cells stratified into a layer or plate. These layers can be categorized according to polarity and adheren properties. Epithelial cells have an apical-basal polarity. It means the apical and basal membranes have different complexes, thus possessing different properties. The invasion of cancer cells from the primary site is called intrainvasion. Afterward, cancer cells should survive in blood circulation and locate the most suitable organ, a secondary site. The cells then invade this organ and proliferate there, as they did at the primary site. This process is referred to as extravasation

In medical terms, metastasis occurs when tumor cells from their primary site invade the basal tissue and enter the bloodstream; once they find an appropriate organ, they attach to it and proliferate. From a biological and biochemical perspective, metastasis includes EMT (epithelial-mesenchymal transition) and MET (mesenchymal-epithelial transition) (Fig. 1).

We must first differentiate between epithelial and mesenchymal cells to better understand metastasis. Epithelial cells exhibit apical-basal polarity and are tightly attached to one another, which is essential for epithelial tissue. A transmembrane glycoprotein E-cadherin is one of the most important signatures of the epithelial cells [5]. As E-cadherin provides cell-cell junctions and polarity in cells, it inhibits cell dispersion [6]. Also, it interacts with α and β catenin through its cytoplasmic domain and organizes the cytoskeleton to keep cells tight [5].

On the other hand, mesenchymal cells have invasion and migration capabilities, and their hallmark gene expression is N-cadherin [5]. They are not uniform and do not have apical-basal polarity or cell-cell tight junctions [5]. The arrangement of actin filaments allows these cells a wide variety of movements. Moreover, they exhibit morphological flexibility [5].

One of the most significant aspects of metastasis is the dynamic nature of mesenchymal and epithelial phenotypes [5]. It means that cells may lose their epithelial phenotype (differentiated cells) and become mesenchymal (undifferentiated cells). For example, during EMT, E-cadherin is suppressed or degraded while N-cadherin is upregulated. EMT is characterized by the loss of E-cadherin expression and induction of N-cadherin [5].

If the phenotype of cancer cells changes from epithelial (attached to their matrix) to mesenchymal (invading their matrix), cancer cells may enter the blood circulation and travel throughout the body in search of the most suitable place for re-proliferation. EMT is required for the invasion stage, while MET is required for re-proliferation. At the secondary site, cancer cells need to change from a mesenchymal to an epithelial phenotype [5]. Cells that undergo EMT are resistant to therapy and show recurrence and metastasis [6].

SNAIL, TWIST, and ZEB are the most important regulators of EMT; they are all transcription factors that suppress epithelial characteristics [5]. Here, we briefly describe the role of SNAIL in EMT. SNAIL expression is linked to poor prognosis in human cancer patients [6]. In general, SNAIL 1 and 2 activities induce aggressive phenotypes [5]. They promote hypermethylation and deacetylation of histones, which in turn suppress epithelial genes like E-cadherin and induce mesenchymal genes such as N-cadherin and MMP-2 and 9 [5–7]. SNAIL1 interacts with other transcription factors like ETS1 to upregulate MMP expression [5].

TGFβ, EGF, HGF, and GSK3β are upstream of SNAILs which can activate them [5]. Besides, SNAIL1 directly activates TWIST and ZEB, which then activates SNAIL in a positive loop [5]. Moreover, HIFα, IKKα, SMAD, NFκB, and STAT3 promote SNAIL1 upregulation at the transcriptional level [7]. Clearly, several molecules contribute to metastasis through SNAIL induction, ranging from growth factors such as EGF to transcription factors such as STAT3.

The following section will discuss anoikis, which is suppressed or inactivated in metastatic cancer cells. What is the relationship between metastasis and anoikis? How can cancer cells inactivate anoikis in order to survive?

## Anoikis

**Fig. 2:**
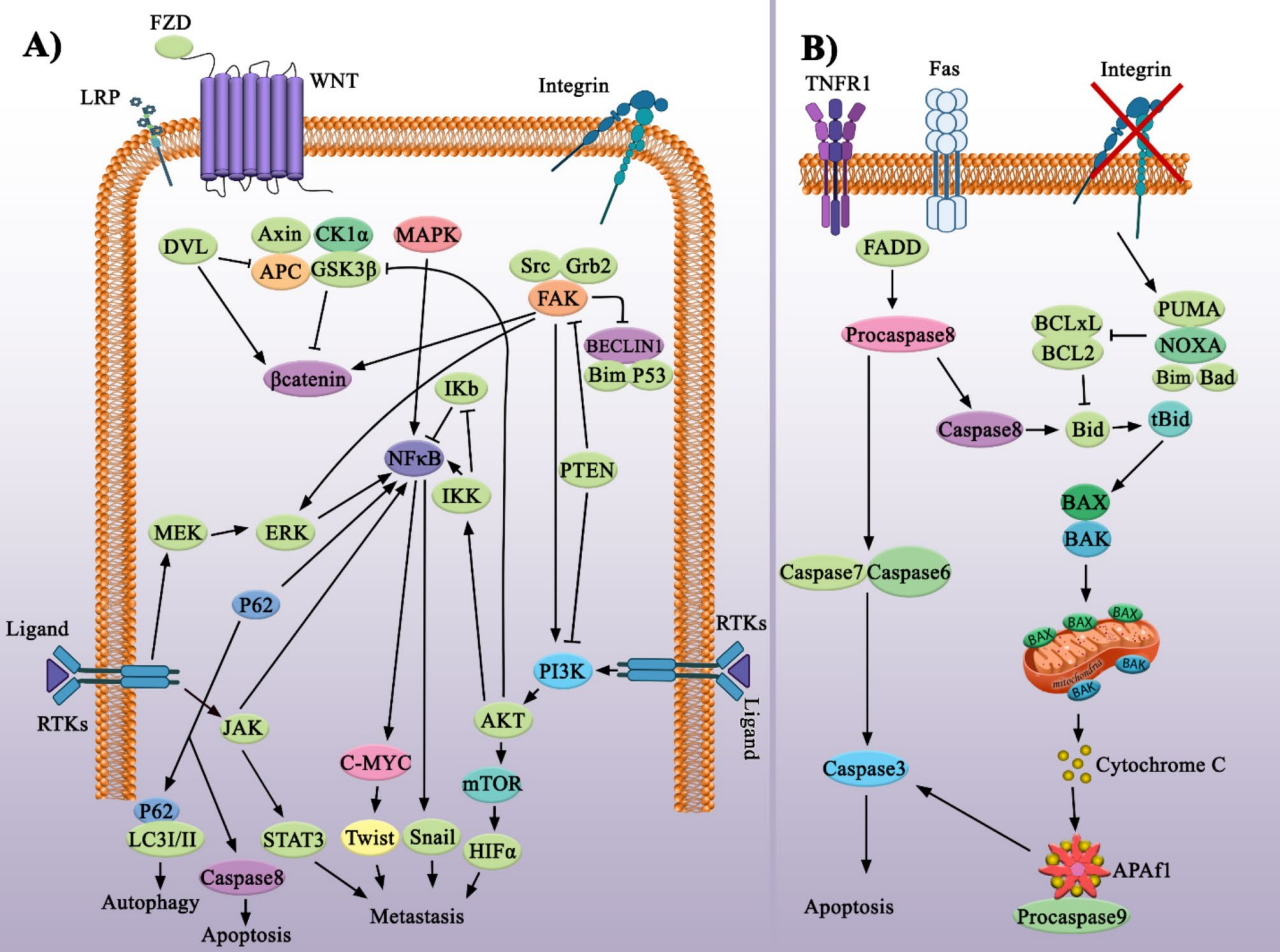
Signaling of metastatic pathways and anoikis **(A)** The signaling of different receptors and molecules like RTKs, FZD, and integrin creates a complex cell network. Integrin downstream signaling is characterized by FAK, which has crosstalks with other pathways such as mTOR and MAPK. In fact, FAK guarantees cell survival. Another interesting crosstalk happens between autophagy and apoptosis. In cancer cells, P62 activates NFκB, which rescues cancer cells from oxidative stress and promotes survival. Meanwhile, P62 regulates apoptosis by activating caspase-8. There are two ways in which autophagy is triggered: one by ATG and LC3 and another by LC3 and P62. In a hypoxic situation, P62 activity is suppressed, and RAS/ERK activity is increased. **(B)** Signalings of programmed cell death

In anoikis, detached cells from the ECM undergo cell death to prevent the proliferation or dispersion of malignant cells [5, 7]. When cells attach to their surrounding ECM, FAK signaling, which promotes cell survival, is activated and involved in BCL2 gene expression [5]. The detachment from the ECM blocks this signaling and triggers both external and internal pathways of anoikis (Fig. 2) [5]. The external pathway of anoikis begins with the binding of the death ligand and death receptor, followed by the recruitment of caspase-8 and the activation of caspase-3 [5]. The internal pathway of anoikis is mediated by BIM, BAD, BIK, BAK, and BAX to form a BAK/BAX complex and block BCL2 anti-apoptotic properties [5]. In both pathways, cytochrome C releases to the cytosol, resulting in the formation of apoptosomes and the activation of effector caspases [5].

Resistance to anoikis is a primary characteristic of EMT, which is possibly mediated by the downregulation of E-cadherin and upregulation of N-cadherin [7]. Further, TWIST, SNAIL, and ZEB confer anoikis resistance to cancer cells by suppressing and inducing E-cadherin and N-cadherin, respectively [7].

## The effect of flavonoids on metastasis and anoikis

There is evidence that flavonoids can block metastasis in different types of cancers by suppressing mesenchymal signature molecules and inducing epithelial ones. Quercetin, apigenin and fisetin suppress SNAIL, TWIST, NFκB, and STAT3 (Table 1). Various cancer cells are susceptible to the effects of these flavonoids. Because flavonoids target multiple molecules from different metastasis pathways, cancer cells are unlikely to develop resistance to these molecules’ signaling.

**Table 1:** The effects of flavonoids on metastatic molecules

Flavonoid	Cancer cell	Decreasing in expression	Inactivation	Increasing of expression or activation	Ref
	Pancreas	MMP9, NFκB	-	-	[46]
	Melanoma	βcatenin, WNT, DVL	-	-	[118]
Fisetin	Colon	-	NFκB, βcatenin	-	[119]
	Blood	-	NFκB, IKK	-	[120]
	Larynge	-	NFκB	-	[47]
Fisetin + erlotinib	Lung	SNAIL	-	E-cadherin	[121]
Fistein + paclitaxel	Lung	SNAIL2, TWIST, MMP2	-	-	[122]
	Breast	VEGF, MMP9	JAK1, STAT3, TWIST	-	[123–126]
	Blood	βcatenin	-	-	[127]
Quercetin	Human teratocarcinoma	-	βcatenin	-	[128]
	Lung	-	SNAIL1/2, TWIST, N-cadherin	E-cadherin	[129]
	Hela	NFκB	-	IKb	[130]
	Colon	-	βcatenin	-	[131]
	Prostate	-	NFκB	IKb	[132]
	Breast	VEGF, MMP9	JAK1, JAK2, STAT3	-	[58, 133]
Apigenin	Lung	MMP2, MMP9, GSK3β	SNAIL1/2, N-cadherin	-	[134, 135]
	Colorectal	AXIN, βcatenin	βcatenin	-	[135, 136]
	Osteosarcoma	βcatenin	-	-	[59]
	Prostate	MMP9	-	-	[137]
	Pancreas	MMP2, MMP7, MMP9, STAT3	STAT3	-	[138]
Luteolin	Glioblastoma	MMP2, MMP9	N-cadherin, βcatenin	E-cadherin	[139]
	Breast	SNAIL1/2, N-cadherin, βcatenin	-	E-cadherin	[64]
	Colorectal	MMP9	-	-	[65]
Silibinin	Bladder	-	GSK3β, βcatenin	-	[56]
Silibinin	Kidney	MMP9	MMP9	-	[140]
Curcumin	Glioblastoma	-	NFκB	-	[141]
Curcumin	Melanoma	-	NFκB, IKK	-	[142]
Butein	Liver	MMP9	MMP9	-	[143]
Butein	Prostate	IKK	NFκB	IKb	[62]
Chalcone	Colon	-	STAT3	-	[63]
Resveratrol	Colorectal	-	NFκB, MMP9, MMP13	-	[144]
Ginger + Gelam honey	Colon	βcatenin, GSK3β	-	-	[145]
Epigallocatechin-3-gallate	Prostate	-	NFκB	-	[146]
Baicalein	Breast	GSK3β	GSK3β	-	[147]
Cardamonin	Breast	GSK3β, VEGF	βcatenin	-	[148]

## Autophagy

Autophagy is a conserved mechanism from yeast to humans and is considered a survival mechanism during times of stress, such as when nutrients are depleted, or growth factors are lost [8–11]. In this process, cytoplasmic components and some organelles are degraded by lysosomes [9]. Autophagy is a survival mechanism for dying cells [12]. It maintains cell homeostasis by degrading aggregated proteins and damaged organelles [6].

Cancer cells also undergo autophagy, just as normal cells do. These cells must increase their level of autophagy in order to survive in stressful conditions such as hypoxia [6]. Studies have demonstrated that autophagy contributes directly to the development of cancer. For example, results show that ATG6 and BECLIN1 are exhausted in approximately 50% of breast, ovary, and prostate cancers [10]. Moreover, deletion of ATG5 and 7 induces liver tumor development in mice models [10].

However, autophagy does not play a direct role in cancer development. In cancer cells, autophagy can either be cytoprotective or cytotoxic. Accordingly, it appears to be contradictory in its effects on cancer development [13]. Data indicate that autophagy inhibits tumor formation in preneoplastic legions [11, 13, 14]. That is, during the early stage of tumor formation, autophagy stabilizes the genome and inhibits oncogenic protein accumulation [13]. On the other hand, results show that in established tumors, the occurrence of autophagy promotes cancer proliferation, growth, survival, and invasion [11, 13, 14]. In fact, autophagy contributes to the resistance of cancer cells against various stressors in the late stages of the tumor [13]. Nonetheless, most research has focused on inhibiting autophagy in cancer therapy [11].

Due to the crosstalk between autophagy and apoptosis, autophagy induction can occasionally trigger apoptosis rather than saving cells [9]. Accordingly, overactivation of autophagy may promote cell death [9]. Autophagy has a threshold, and the induction of death or survival by autophagy depends on autophagy duration [15]. The long-term duration of autophagy consumes many cytoplasmic components, resulting in cell death [9]. Besides, autophagy might degrade some survival factors and promote cell death [9]. Molecules involved in the machinery of autophagic cell death may be different from those involved in the survival process [9].

For a deeper understanding of the role of autophagy in cell death, it is necessary to examine the interaction between anti-apoptotic and pro-apoptotic molecules with BECLIN1, one of the major players in autophagy. The first step is to examine BECLIN1 interactions with anti-apoptotic molecules. BECLIN1 has an ECD, enabling it to mediate autophagy [16]. However, BECLIN1 is not a pro-apoptotic molecule even when it is overexpressed in the cells [16]. In some cancer cells, it is located at the plasma membrane, cytoplasm, and nucleus [16]. Some anti-apoptotic family members of BCL2 interact with BECLIN1 [16]. Studies show that the BECLIN1/BCL2 interaction has a regulatory role in autophagy [17] because BCL2, MCL, and BCLxl are BECLIN’s inhibitors [6, 16]. As a result, BCL2, which is overexpressed in many cancers, blocks apoptosis and autophagy [6]. It is important to note that BECLIN1 cannot block BCL2 activity in mitochondria, and it can only co-localize at the inner membrane of mitochondria with BCLxl [16].

BECLIN1 and pro-apoptotic molecules interact with each other. BAD, NOXA, PUMA, and BIK can induce autophagy [16]. When NOXA overexpression induces cell death, it detaches BECLIN1 from MCL and triggers autophagy [12]. P53 has a dual role in autophagy. In the nucleus, p53 facilitates autophagy while inhibiting autophagy in the cytoplasm [11]. During ER stress, unfolded proteins induce autophagy and apoptosis by activating caspase-3 and 10 in cancer cells [18]. In cancer cells, caspase-3 cleaves BECLIN1, which induces apoptosis and inhibits autophagy [17]. Caspase cleavage might disrupt BECLIN1/BCL2 complex [17]. Moreover, studies have found that caspases-3, 7, and 8 cleave BECLIN1 during apoptosis in Ba/F3 cells [19]. BECLIN1 fragments cannot induce autophagy. They accumulate in mitochondria, promoting cytochrome C release and apoptosis [19].

## The effect of flavonoids on autophagy

As mentioned earlier, the duration and intensity of initial stress can cause differences between cytotoxic and cytoprotective autophagy in cancer cells [20]. Hence, flavonoids may induce autophagy in cancer cells by triggering various stress responses, such as ER stress. For example, curcumin induced cytotoxic autophagy in melanoma and glioma cancer cells [21, 22]. In glioma cells, it may induce G2/M cell cycle arrest, inhibit mTOR pathway activity and increase ERK pathway activity [22]. Cytotoxic autophagy, induced by curcumin, was dampened when the ERK pathway was inhibited or the mTOR pathway was activated [22].

In thyroid cancer cells, apigenin also induced G2/M cell cycle arrest and autophagic cell death [23]. Apigenin decreased P62 expression and increased BECLIN1 in these cells [23]. Apigenin caused autophagy in colorectal cancer cells by reducing βcateinin and suppressing the mTOR pathway [24].

Activation of AMPK and ERk by resveratrol induced cytotoxic autophagy in leukemic cancer cells, which in turn activated P62[20]. Resveratrol increased both mRNA and protein levels of P62 in these cells [20]. In this situation, P62 binds to LCI and II directly and promotes autophagy [20]. A similar effect of resveratrol has been observed on ovarian cancer cells. In these cells, resveratrol induced the expression of P62, LCII, and caspase-3, which led to apoptosis [25].

Research has shown that fisetin has cytotoxic and cytoprotective effects on cancer cells. In prostate cancer cells, fisetin caused autophagic cell death, and the suppression of BECLIN1 decreased fisetin-induced death [26]. On the other hand, a study demonstrated that fisetin induced cytoprotective autophagy in pancreas cancer cells through ER and mitochondrial stress [27].

Silibinin inhibited ERα activity, resulting in autophagy induction followed by apoptosis in breast cancer cells [28]. Silibinin increased P62 activity in renal cancer cells, causing cytotoxic autophagy and anti-metastatic effects [29]. Silibinin and fisetin in renal and prostate cell lines suppressed mTOR and cytotoxic autophagy in these cells, respectively [26, 29].

Because flavonoids can activate caspases like caspase 3, these activated caspases are likely to cleave ATG4D or BECLIN1 and cause apoptosis. The presence of flavonoids can also potently impose stress on cancer cells, thus activating cytotoxic responses. Since flavonoids activate AMPK and deactivate mTOR pathways, it is also possible that they can induce a significant shock in cancer cells, resulting in apoptosis that cannot be prevented by cytoprotective autophagy.

## Autophagy and metastasis dynamic

Autophagy provides the energy required for the survival and migration of cancer cells during metastasis [8]. Autophagy alters the cell-cell junction by inhibiting E-cadherin and promoting cancer cell invasion and migration [8].

The factors that induce autophagy are similar to those that initiate metastasis, such as hypoxia, detachment from the ECM, and nutritional deficiency [30]. TGFβ and hypoxia can trigger EMT through autophagy [30]. The ULK2 phosphorylation of BECLIN1 in the primary complex can induce EMT and invasion by down-regulating E-cadherin [30]. As an autophagy cargo adaptor, P62 binds to TWIST, preventing its proteasomal degradation; therefore, P62 maintains a mesenchymal phenotype [30].

Contrary to these studies, there is a lack of consensus concerning the relationship between autophagy and metastasis. Indeed, data suggest that autophagy’s promotive or inhibitory role in metastasis depends on metastasis status. While autophagy suppresses metastasis in the early stages, it facilitates metastasis in the late stages by promoting cancer cell survival and colonization [10, 13, 31].

It is important to note that the effect of autophagy on metastasis varies according to the type of tumor. For example, autophagy can contribute to the invasion and spread of liver cancer. In vitro studies have shown that a lack of nutrients induced autophagy and activated EMT in liver cancer cells [32]. ATG-3 and 7 deletions suppressed EMT and invasion in these cells [32]. However, in glioblastoma cancer cells, autophagy blocks invasion and metastasis. In these cells, starvation or rapamycin-induced autophagy reduced invasion, migration, and expression of SNAIL1 and 2 [32]. On the other hand, migration and invasion were increased by inhibiting BECLIN1, ATG-5, or 7 [32].

Following is a discussion of several significant pathways involved in metastasis and their inhibitors in cancer treatment.

### WNT/ βcatenin pathway in cancer cells

The WNT canonical pathway is a conserved pathway involved in proliferation, growth, differentiation, apoptosis, migration, invasion, and metastasis [33]. Uncontrolled activation of the WNT pathway may promote the progression and development of various cancers [33]. A dysfunction in βcatenin, a transcription factor that is central to this pathway, could result in tumorigenesis [33]. Accordingly, it is highly regulated. Without WNT, βcatenin is attached to cytoplasmic cadherins and would not translocate to the nucleus [34]. GSK3β and CK1α form a complex that phosphorylates βcatenin and promotes proteasomal degradation [33, 34]. WNT/FZD binding activates this signaling pathway. After that, DVL recruits GSK3β, AXIN, APC, and CK1 and forms a complex [33]. Phosphorylation and inactivation of GSK3β increase the βcatenin cytosolic level [33]. Unphosphorylated βcatenin translocates and accumulates in the nucleus, promoting C-MYC and cyclin D transcription [33].

GSK3β regulates the cytoplasmic level of SNAIL2, and active WNT inhibits GSK3β activity which stabilizes SNAIL2 and promotes EMT [35]. It can be concluded that there is a direct correlation between the WNT signaling pathway and metastasis. In overactivated WNT signaling in cancer cells, when PI3K/AKT signaling is blocked, βcatenin accumulates in the nucleus and promotes metastasis [35].

There are many inhibitors for every component of this pathway in cancer therapy. Suppression of βcatenin or WNT blocked cancer cell proliferation in animal models [35]. LGK974 and IWP that block WNT secretion reduced tumor size in head and neck cancer animal models and suppressed the migration capability of breast cancer cell lines, respectively [36, 37]. Moreover, OMP18R5 is a monoclonal antibody that targets FZD-inhibited tumor growth in animal models [38].

### JAK/STAT3 pathway in cancer cells

In the resting state, STAT3 is inactive and located in the cytoplasm [39]. STAT3 dimerizes when it is activated by stimulators such as EGFR, VEGFR, PDGFR, CSF1, and JAK. Then, it translocates to the nucleus and engages in transcriptional activity [39–41]. The dysregulated function of STAT3 has been observed in many cell lines and tumor tissues [39, 41]. Abnormal STAT3 activation causes proliferation, survival, invasion, angiogenesis, and metastasis [39]. Constitutive activation of STAT3 is positively correlated with the expression of C-MYC, cyclin D, BCL2, BCLxl, MCL1, SURVIVIN, CIAP, and MMPs, while it is negatively correlated with the expression of P53 [39, 41]. As a result, it promotes cell proliferation by upregulating cyclin D and promotes cell survival by upregulating anti-apoptotic molecules such as BCL2. Moreover, STAT3 facilitates metastasis by enhancing the expression of MMPs [39]. Studies suggest that STAT3 activation is enough for malignant cell transformation [39]. There is a correlation between STAT3 overactivation and poor prognosis in several types of cancer [40, 41].

Targeting STAT3 is a cancer therapy strategy. Experimental and animal studies indicate that STAT3 inhibition prevents tumor growth and metastasis [39]. In vitro and in vivo studies have demonstrated that the SH2 domain inhibitors CJ-1383, S31-201, and BP-1-102 can induce apoptosis and show anti-tumor activity, respectively [40, 41].

### IKK/NFκB pathway in cancer cells

Active NFκB is linked to poor prognosis, low survival rate, and metastasis in cancer patients [42]. NFκB regulates various genes involved in cancer cells’ survival, invasion, and angiogenesis [42]. Constitutive expression of NFκB in cancer cells results in resistance to chemotherapy-induced apoptosis [42]. NFκB is a transcription factor that induces EMT by regulating SNAIL1/2, TWIST gene expression [5, 7, 43]. NFκB can confer chemotherapy or radiotherapy resistance on cancer cells by inhibiting TRAF1 (Tumor necrosis factor receptor (TNFR) associated factor 1) and other mechanisms of cell death [5].

When TNF activates NFκB, it blocks autophagy in cancer cells, and NFκB inhibition re-activates autophagy. NFκB regulates BECLIN1, but its role in autophagy remains unclear [16]. On the other hand, inhibition of NFκB- dependent autophagy in cancer cells sensitizes them to apoptosis [7]. NFκB confers anoikis resistance because NFκB targets genes like CIAP2, survivin, BCL2, BCLxl, and XIAP, which make cancer cells resistant to anoikis [43].

Studies have shown that NFκB inhibitors such as narasin, fluorosalan, and emetine could inhibit IB and NFκB in cervical cancer cells. Besides, these agents suppressed Hela cell growth significantly [44, 45].

It is also worthwhile to note that flavonoids influence these pathways (Table 1). They can potentially eradicate cancer cells by inhibiting STAT3, NFκB, and βcatenin. There are complex interactions between molecules that are involved in autophagy, apoptosis, anoikis, and metastasis, which makes cancer therapy challenging.

Results of in vitro studies serve as a basis for further research in animal settings. Is it possible for flavonoids to serve as anti-cancer agents in vivo? The following section examines the effects of flavonoids in animal studies.

### The effect of flavonoids on different types of tumors in animal model studies

Fisetin in animal model of melanoma [46], laryngeal [47], prostate [48], and colorectal [49] inhibited tumor growth and reduced tumor weight. In prostate cancer, mice were athymic, and cytotoxic T-cells had no role in tumor regression. Also, fisetin and sorafenib had synergistic anti-tumor effects on tumor growth in vivo studies [50].

Quercetin can inhibit tumor initiation, progression, and invasion of different tumor types. For example, quercetin inhibited the initial liver tumorigenesis in vivo studies and promoted apoptosis by increasing BAX/BCL2 ratio [51]. Moreover, in an animal study, quercetin prevented angiogenesis and proliferation in prostate tumors [52]. Quercetin in SCID mice with breast cancer xenografts reduced tumor growth by almost 70% [53]. It also blocked metastasis in pancreas tumors [54]. It was observed that quercetin reduced tumor size and number in mice models of skin cancer [55].

Silibinin decreased invasion, migration, and angiogenesis in several tumor types [56]. For example, it blocked βcatenin signaling in bladder tumors and hindered metastasis [56]. In renal tumors, silibinin inhibited tumor growth [57]. A study found that apigenin suppressed AKT and stimulated JNK in leukemia in an animal prostate tumor model [58]. Apigenin significantly decreased VEGF and HIFα expressions in lung tumors (nude mice model) [58]. Besides, apigenin induced apoptosis in the osteosarcoma xenograft model [59]. Curcumin inhibited glioma and melanoma growth in vivo studies [22, 60]. In the mesothelioma xenograft model, curcumin blocked NFκB nucleus localization [61].

Butein and chalcone inhibited prostate and Ehrlich tumor growth in athymic mice, respectively [62, 63]. Luteolin blocked β catenin signaling in the lung xenograft model [64] and prevented metastasis in the lung [64], colorectal [65], and TNBC tumors [66]. Luteolin increased cisplatin’s anti-tumor effects on ovary tumor xenografts, induced apoptosis, and inhibited tumor growth [67, 68].

**Fig. 3:**
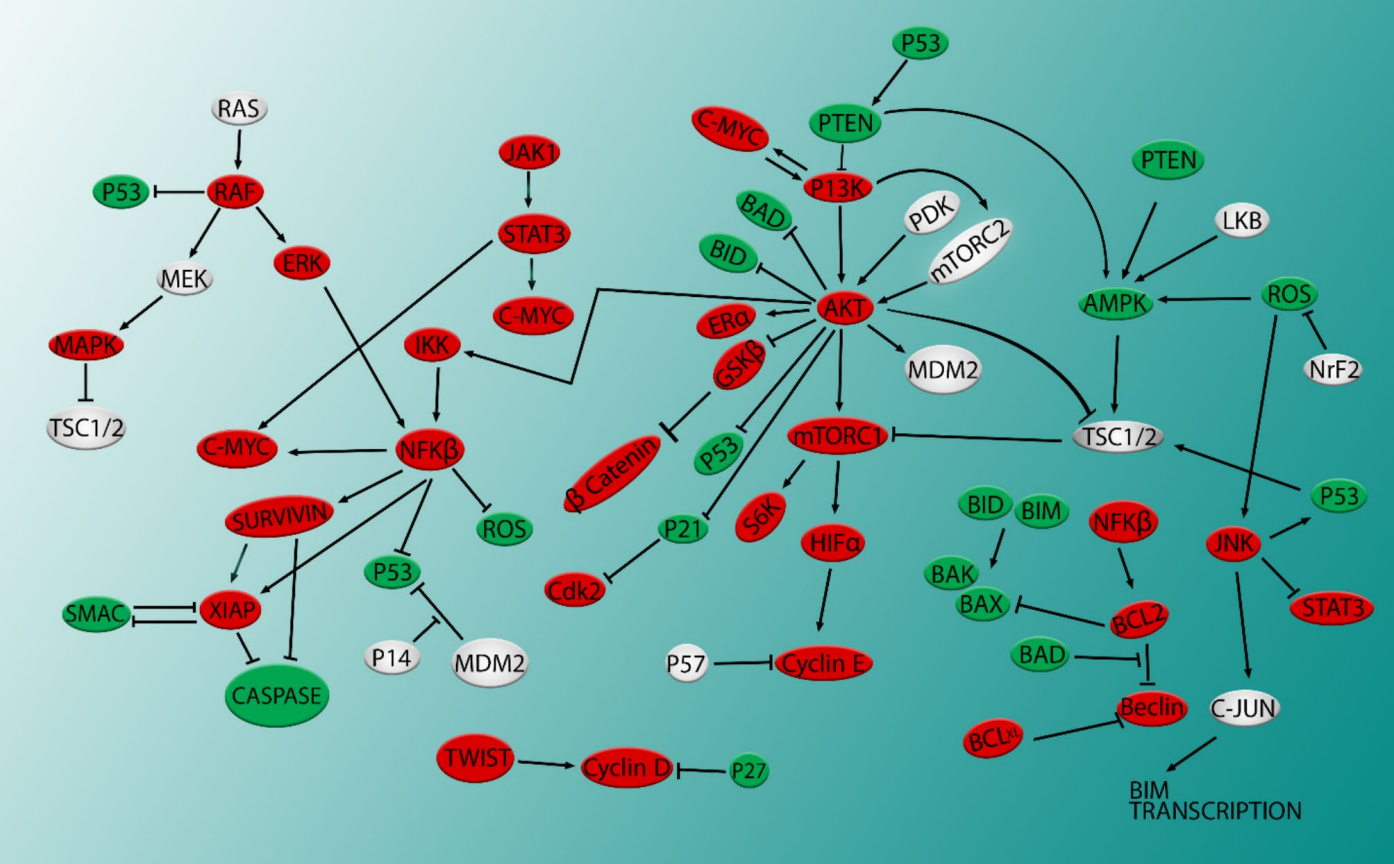
Flavonoids target panel of molecules

All affected molecules are shown in the following map, indicating that flavonoids can suppress anti-apoptotic and oncogenic molecules and simultaneously activate different pro-apoptotic molecules (Fig. 3).

Although this map may appear complex at first glance, it is a simplified version of a real signaling map within a cell. Figure 3 illustrates various molecules involved in processes like growth, survival, proliferation, apoptosis, anoikis, metastasis, and autophagy. Red molecules are suppressed or inactivated by flavonoids, whereas green molecules are induced or activated by them. The map was prepared based on the results of cancer studies. Flavonoids have the potential to be successful in the treatment of cancer. As flavonoids target a wide range of molecules from different processes, they prevent cancer cells from compensating, so they are no longer resistant to therapy.

### Selectivity of flavonoids

In vitro and in vivo studies strongly suggest flavonoids may have therapeutic effects on cancer. In addition to targeting specific molecules in different kinds of cancer cells, flavonoids offer many significant advantages as anti-cancer agents. Flavonoids also target cancer cells selectively. It means they have little or no effect on normal organs and only eliminate tumors. Several in vitro studies have demonstrated that different flavonoids target cancer cells selectively [22, 69–72]. In fact, xanthohumol, fisetin, curcumin, and EGCG only target cancer cells from BC, prostate, glioma, and colon, respectively [22, 69, 70, 73].

Moreover, clinical data indicate that flavonoids exert little or no toxicity on normal organs. For example, xanthohumol had no side effects on normal organs [74] and no change in body metabolism in animal models [75, 76]. Furthermore, EGCG, was well tolerate in prostate [76, 77], head and neck [78], breast [79], ovary [80], and lung [81] cancer patients in clinical trials [82].

Following is a summary of studies conducted on human cancer patients in various settings. Results from human studies are consistent with those obtained in vitro and in vivo.

**Preclinical and clinical (**in vivo**) studies of flavonoids in cancer therapy**.

Various studies investigated the effects of flavonoids as part of the diet, supplement, single treatment, or co-treatment in different cancers. This part reviews preclinical and clinical studies of flavonoid cancer therapy.

### Flavonoids as an injectable medicine

#### Single-treatment

In some studies, flavonoids are administered as a single cancer therapy treatment. A study on 24 xenograft lung cancer (A549 cells) divided the model into three groups. The control group received normal saline at 10 ml/kg/d. The second group received cisplatin at 4 mg/kg/d, and the third group received epigallocatechin-3-gallate (EGCG, a major flavonoid in green tea) at 50 mg/kg/d (n = 8 and Intraperitoneal injection for all groups). EGCG treatment reduced tumor size but to a lesser extent than cisplatin treatment (37% vs. 50%). However, toxicity measured by mice weight was lower in the EGCG group than in the cisplatin group. EGCG increased apoptotic pathways by induction of activated caspase-3 and Ku70 expression reduction. The low toxicities and side effects of EGCG make it a promising chemotherapy agent [83].

In another study focusing on the synergistic effects of anthocyanidins, one of the major subclasses of flavonoids, anti-cancer properties were examined. In a xenograft model, eight-week treatment with a native anthocyanidin mixture or delphinidin extracted from blackcurrant and bilberry suppressed tumor growth in both interventional groups (60–65%). The prescription dose of delphinidin was 3 times higher than the mixture, suggesting that anthocyanidins have a synergistic effect in preventing tumor growth [84]. In the xenograft model of breast cancer, Oncamex, an analog flavonoid (myricetin-derived), was administered intraperitoneally for 14 days and inhibited tumor growth, viability, and Ki-67 expression (a proliferation marker) [85]. According to Chien et al., 35 days of intraperitoneal treatment of apigenin inhibited the growth and metastasis of prostate cancer cells in animal models [86]. In a pancreatic cancer model, injection of EGCG intraperitoneally for 10 days suppressed tumor growth and metastasis in a dose- and time-dependent manner [87].

#### Co-treatment

First-line cancer treatments include radiation therapy and chemotherapy [88]. One of the most challenging aspects of cancer therapy is resistance to treatment [89]. In some studies, flavonoids were used as co-treatment with chemo/radiotherapies to reduce resistance or increase sensitization (as a synergist). On the other hand, flavonoids protect normal cells from these invasive therapies due to their higher specificity for cancer cells than normal cells [87, 90]. In a 28-day study, quercetin injection intraperitoneally reversed docetaxel resistance in mice with metastatic prostate cancer. It was found that, due to drug resistance, tumor size and weight in the docetaxel group were not different from those in the control group. However, tumor growth was more rapid in the quercetin group and most effective when used in combination (docetaxel + quercetin). The proliferation of tumor cells and modulation of apoptosis pathways were observed only in the quercetin and combination groups but not in the docetaxel group [91].

Using an intraperitoneal injection every two days, Huang et al. administered 2 mg of apigenin (flavonoid) and 30 mg of abivertinib (chemo drug) to 4 xenografted mice models of B-cell lymphoma. After 10 days of intervention, they noted a 32.5%, 48%, and 80% reduction in tumor mass in the apigenin, abivertinib, and combination groups, respectively. Also, a combination of apigenin and abivertinib was more effective at reducing tumor size than either agent alone. Induced apoptosis and impeded proliferation were the proposed mechanisms. However, animal survival was not checked [92]. Injection of apigenin may help cell death as a radiosensitizer in Ehrlich carcinoma murine model. It may also reduce the γ- irradiation dosage [93]. A study by Lin et al. also demonstrated that quercetin and radiotherapy increased tumor cell death in the xenograft model of colorectal cancer [94]. Additionally, radiotherapy is insufficient to deal with cancer cells’ oxidative stress and apoptosis. In the context of cancer radiotherapy, flavonoids can act as a co-inducer of ROS in cells and increase cell death [90].

There has been no research on the short-term and long-term effects of flavonoids. Despite the high plasma concentrations of flavonoids after dietary consumption, high doses of flavonoids were used.

#### Flavonoids as oral medicine

In an in vivo study, Navarra et al. examined the effects of bergamot juice (BJe) on a colorectal cancer model. A flavonoid-rich bergamot juice extract (BJe) was mixed into the diet of Pirc rats (representative of colorectal cancer). The extract contained 35–70 mg of flavonoids per kilogram of body weight. After 12 weeks of intervention, the colon preneoplastic lesions mucin-depleted foci (MDF) reduced in a dose-related manner. Rats supplemented with 70 mg/kg BJe showed a reduction in the number of tumors [95].

The treatment of 20 men with biochemically recurrent prostate cancer was conducted in a phase II study using 141 mg of soy isoflavone per day for 12 months. The study showed reduced serum PSA from 56 to 20% as a recurrent prostate cancer index [96]. Another phase II study examined the effects of EGCG treatment at a dosage of 2000 mg twice daily for 6 months in patients with early-stage chronic lymphocytic leukemia (CLL). Results indicated that absolute lymphocyte count (ALC) and lymphadenopathy declined significantly [97].

While in vitro studies have shown the positive effects of flavonoids on the health system, their use as a therapeutic agent in vivo and clinical trials may face various challenges, particularly when administered orally. Even though flavonoids are found in low concentrations (micrograms to milligrams per kg of plant mass) [98], the key challenge is limited bioavailability produced by the physicochemical features of flavonoids, which leads to low absorption, rapid metabolism, and exertion in the body [1, 98, 99].

Almost all flavonoids, except catechins, are bonded to a sugar molecule in the form of “glycosides,“ which are less absorbable than free “aglycones.“ As a result, they are less bioavailable because intact glycosides cannot enter small intestine cells and need to be deglycosylated before intestinal uptake. However, some exceptions exist, such as quercetin, where the glycoside form absorbs more than the aglycone form. The structure and position of the sugar moiety also cause diversity in flavonoid absorption [100, 101].

Improving the bioavailability of flavonoids makes these herbal compounds more impressive in the food supplements and pharmaceutical industry. Using nano-delivery systems (nanocarriers) is one method to promote flavonoid bioavailability [102, 103]. For example, when quercetin was administered orally to rats, bioavailability increased from 3.61 to 23.58% with nanosuspension [104]. A murine study found that oral administration of naringenin as nanoparticles had a 96-fold higher bioavailability than the free form of NRG [105]. Also, the daidzein–lecithin complex in mixed micelles showed a 9-fold increase in the oral bioavailability of free daidzein in rats [106]. However, other aspects of nanocarriers, such as cytotoxicity, should be considered [103]. Another way to improve biological value is microemulsion formulation to increase absorption [107] and transporting ability [108]. In addition, the bioavailability of flavonoids can be improved by methylation [109], metal ions conjugation, and a radiation-modified structure [110].

### Advantages and disadvantages of flavonoids in cancer therapy

Here we outline the potential advantages and disadvantages of flavonoids as natural products consumed by our bodies on a daily basis.

Advantages.

Several advantages of polyphenolic compounds include their beneficial biological roles in human health, including antioxidant, anti-inflammatory, anti-carcinogenic, and immune-modulating properties, their easy availability from a wide range of food resources such as fruits, vegetables, tea, and beans and their apparent safety profile in high doses (140 g/day) [1, 111, 112].

Disadvantages.

Flavonoids (e.g., catechins, epigallocatechin-3-gallate, quercetin) act as iron chelators with antioxidant properties, which can adversely affect iron deficiency and homeostasis depending upon their dose [113]. Furthermore, flavonoids (e.g., hesperidin, quercetin, catechins) can inhibit the function of enzymes, particularly digestive enzymes, which may be unfavorable for athletes, older individuals, or those with enzyme problems [113, 114]. More importantly, some flavonoids (e.g., myricetin, apigenin, kaempferol, and isoquercitrin) can influence intestinal microbiota–an antimicrobial effect–in a dose-dependent manner [113, 115]. However, there were also some controversial findings. There is evidence that flavonoids (e.g., quercetin, naringenin, and resveratrol) can adversely affect the metabolism of drugs [113]. Besides, high intake of flavonoids can cause DNA damage and show genotoxicity, in specific circumstance [116]. It is imperative to be aware of the interactions of polyphenolic compounds with drugs, especially in patients with sensitive therapeutic indices.

Thanks to the in vitro studies, there is a wide variety of data about flavonoids, but translating the results into the human body system is challenging. Overall, flavonoids should be used according to specific circumstances [117].

## Conclusion

Numerous studies reveal that flavonoids have anti-cancer effects. These natural products can induce apoptosis and autophagic cell death, inhibit proliferation and metastasis, and overcome drug resistance in cancer cells. Results have been reported in both in vitro and in vivo contexts. One of the interesting characteristics of flavonoids is that, in some cases, they can selectively target tumor cells, thereby preventing their cytotoxic effects on normal cells. Further, they have an extensive range of targets, meaning they can suppress or induce the expression of various molecules involved in apoptosis, autophagy, metastasis, and proliferation. This property enables flavonoids to overcome resistance. More specifically, flavonoids affect several molecules involved in different processes, such as apoptosis and proliferation, and prevent cancer cells from developing compensatory signaling to become resistant. Therefore, using flavonoids in cancer therapy would eliminate a major obstacle to cancer therapy, and a growing body of research indicates flavonoids have the potential to treat cancer.

## Electronic supplementary material

Below is the link to the electronic supplementary material.

Supplementary Material 1

## Data Availability

Not applicable.
