# Supplementary material for: Flavonoids target different molecules of autophagic and metastatic pathways in cancer cells
Source: Cancer Cell Int. 2023 Jun 12;23:114. doi: 10.1186/s12935-023-02960-4 (PMC10262367; doi:10.1186/s12935-023-02960-4)
Supplement: Supplementary file 1 — Supplementary Material 1 [file 12935_2023_2960_MOESM1_ESM.docx]

MAPK: Mitogen-activated protein kinase, EGF: Epidermal growth factor, EGFR: Epidermal growth factor receptor, HRG: Heregulin, DVL: Dishevelled, IGF: Insulin-like growth factor, IGFIR: Insulin-like growth factor 1 receptor, GSKβ: Glycogen synthase kinase β, HER2: human epidermal growth factor receptor 2, HER3: human epidermal growth factor receptor 3, JAK: Janus Kinase, STAT: Signal Transducer and Activator of Transcription, JNK: c-Jun N-terminal Kinases, XIAP: X-linked inhibitor of apoptosis protein, PKC: Protein kinase C, PI3K: Phosphoinositide 3-Kinases, AKT: Protein kinase B known as AKT, mTOR: mammalian target of rapamycin or mechanistic target of rapamycin, mTORC1: mTOR complex 1, mTORC2: mTOR complex 2, PARP: Poly (ADP-ribose) polymerase, CDK: Cyclin-dependent kinases, CDC: cell division cycle protein , ER: Estrogen receptor, ERK: Extracellular signal-regulated kinase, PTEN: Phosphatase and tensin homolog, Rb: Retinoblastoma, BAX: Bcl-2-associated X protein, BAK: Bcl-2 homologous antagonist/killer, BIM: Bcl-2 Interacting Mediator of cell death, BID: BH3 interacting domain death agonist, BCL2: B-cell lymphoma 2, NADPH: Nicotinamide adenine dinucleotide phosphate, AMPK: 5' AMP-activated protein kinase, ATP: Adenosine triphosphate, NFκB: Nuclear Factor Kappa B, SCID: Severe combined immunodeficiency, PDFGR: Platelet-Derived Growth Factors Receptor, GLUT: Glucose transporter, MMP: Matrix metalloproteinase, IRS: Insulin receptor substrate, VEGFR: Vascular endothelial growth factor receptor, S6K: S6 Kinase, VEGF: Vascular endothelial growth factor, TSC: tuberous sclerosis complex, DR: Death receptor, TNBC: Triple negative breast cancer, MEK: mitogen-activated protein kinase, ROS: Reactive oxygen species, HIF: Hypoxia Inducible Factor, IKB: NF-kappa-B inhibitor. RTK: Receptor tyrosine kinase, 4EBP: factor 4E (eIF4E)-binding protein, RAF: rapidly accelerated fibrosarcoma, RAS: Rat sarcoma virus, FDA: The United States Food and Drug Administration, ELK1: ETS Like-1 protein, FZD: Frizzled, AP1: Activator protein 1, ZEB: Zinc finger E-box-binding homeobox, SAMC/DIABLO: Second mitochondria-derived activator of caspase/direct inhibitor of apoptosis-binding protein with low pI (Smac/DIABLO), APAF: Apoptotic protease activating factor, TNF: Tumor necrosis factor, TRAIL: TNF-related apoptosis inducing ligand, FADD: Fas-associated death domain, TRADD: Tumor necrosis factor receptor type 1-associated DEATH domain, DISC: death-inducing signaling complex, MDMD2: murine double minute 2, MCL: Myeloid cell leukemia, IAP: Inhibitor of apoptosis, PUMA: p53 upregulated modulator of apoptosis, LKB: Liver Kinase B, PDK: phosphoinositide-dependent protein kinase, IF4B: initiation factor 4B, HSP: Heat shock protein, IKK: inhibitor of nuclear factor-κB (IκB) kinase, FAK: Focal adhesion kinase, ECM: extracellular matrix, Atg: Autophagy-related, ULK: Unc-51 like autophagy activating kinase, TGF: Transforming growth factor, WNT: Wingless-related integration site, CSF: colony stimulating factor, LC: Microtubule-associated protein 1A/1B-light chain,
